# Morphological variability of *Histershanghaicus* Marseul, 1862 (Coleoptera, Histeridae)

**DOI:** 10.3897/BDJ.13.e151478

**Published:** 2025-05-20

**Authors:** Ji-Huan Zheng, Tomáš Lackner

**Affiliations:** 1 Guangdong Key Laboratory of Animal Conservation and Resource Utilization, Institute of Zoology, Guangdong Academy of Sciences, Guangzhou, China Guangdong Key Laboratory of Animal Conservation and Resource Utilization, Institute of Zoology, Guangdong Academy of Sciences Guangzhou China; 2 Department of Environmental Systems Science, ETH Zürich, Zürich, Switzerland Department of Environmental Systems Science, ETH Zürich Zürich Switzerland

**Keywords:** dorsal elytral striae, morphological variability, distribution

## Abstract

Dorsal elytral striae serve as key diagnostic characters in the taxonomy and species identification of Histeridae beetles. The variation of dorsal elytral striae in *Histershanghaicus* Marseul, 1862 is documented herein, based on specimens collected from Gutian Nature Reserve (Guangdong Province) and Jiulianshan Nature Reserve (Jiangxi Province), southern China. The distribution of *H.shanghaicus* is also updated.

## Introduction

It is widely recognised that the presence and configuration of dorsal elytral striae serve as important diagnostic characters in taxonomy and identification of Histeridae (e.g. Bickhardt 1910 or Ôhara 1989). However, the variation in dorsal elytral striae has been observed in several histerid species within the genera *Hister* Linnaeus, 1758, *Margarinotus* Marseul, 1853, *Platylister* Lewis, 1892 etc. For example, the species *Histerjavanicus* Paykull, 1811 has elytral striae that exhibit a wide morphological gradient, ranging from four complete dorsal and nearly complete sutural elytral striae, to merely two complete dorsal and a strongly reduced or even absent sutural elytral stria (Mazur 1985, Ôhara 1989).

*Histershanghaicus* Marseul, 1862 occurs in southern and south-eastern China (including Shanghai, Zhejiang, Fujian, Guangdong Provinces), Laos and Vietnam (Mazur 2011b). The typical ‘*shanghaicus*’ specimen features dorsal elytral striae I–II complete and basally abbreviated stria III. Normally, sutural elytral stria is absent in this species (Mazur 2011a, Zhou et al. 2021).

Here, the variation of the dorsal elytral striae of *Histershanghaicus* specimens collected from Gutian Nature Reserve (Huizhou, Guangdong Province) and Jiulianshan Nature Reserve (Longnan, Jiangxi Province), southern China, is reported.

## Materials and Methods

Specimens were collected by flight intercept traps (Nie et al. 2017) between 2020 and 2021 and preserved in 99% ethanol before proceeding to morphological examination. Morphological characters were examined with a Zeiss Stemi 305 microscope. Male genitalia were dissected using the following procedure: the abdomen was removed from each specimen, boiled in water for about five minutes and then transferred to a vial containing 10% potassium hydroxide (KOH) solution for approximately two hours. The abdomen with the aedeagus was then washed in distilled water three to four times and subsequently transferred to a cavity slide using fine forceps. The aedeagus was separated from the abdomen using a hooked fine dissecting needle. The terminology used in this study follows Ôhara (1994). Habitus images were taken using a SONY a7 digital camera. Aedeagal images were taken using a Nikon D610 digital camera, attached to a Zeiss V/A1 microscope (with 5× objective lens). A cable shutter release was used to prevent the camera from vibration. All images were stacked using Helicon Focus 7 to obtain the full depth of focus and the resulting output was edited with Adobe Photoshop 2025. The specimens were deposited in Institute of Zoology, Guangdong Academy of Sciences, Guangzhou, China (GIZ).

## Results

**Figure 1.**
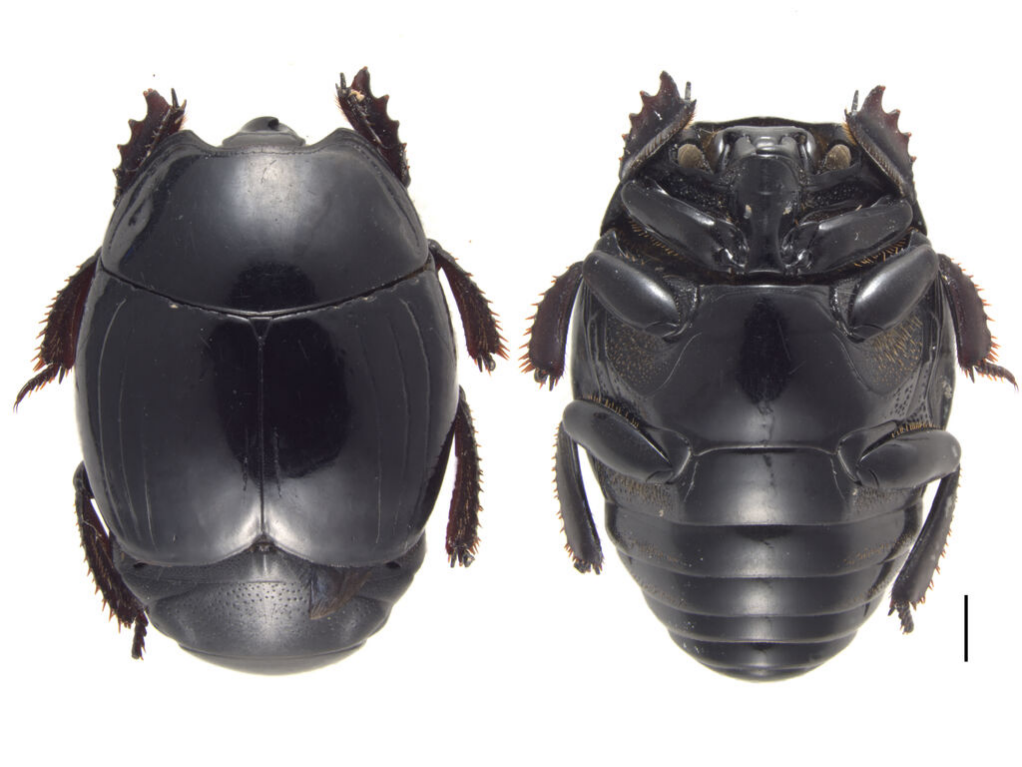
Habitus of *H.shanghaicus* Marseul, 1862 from dorsal (left) and ventral (right) view. Scale bar = 1 mm.

**Figure 2.**
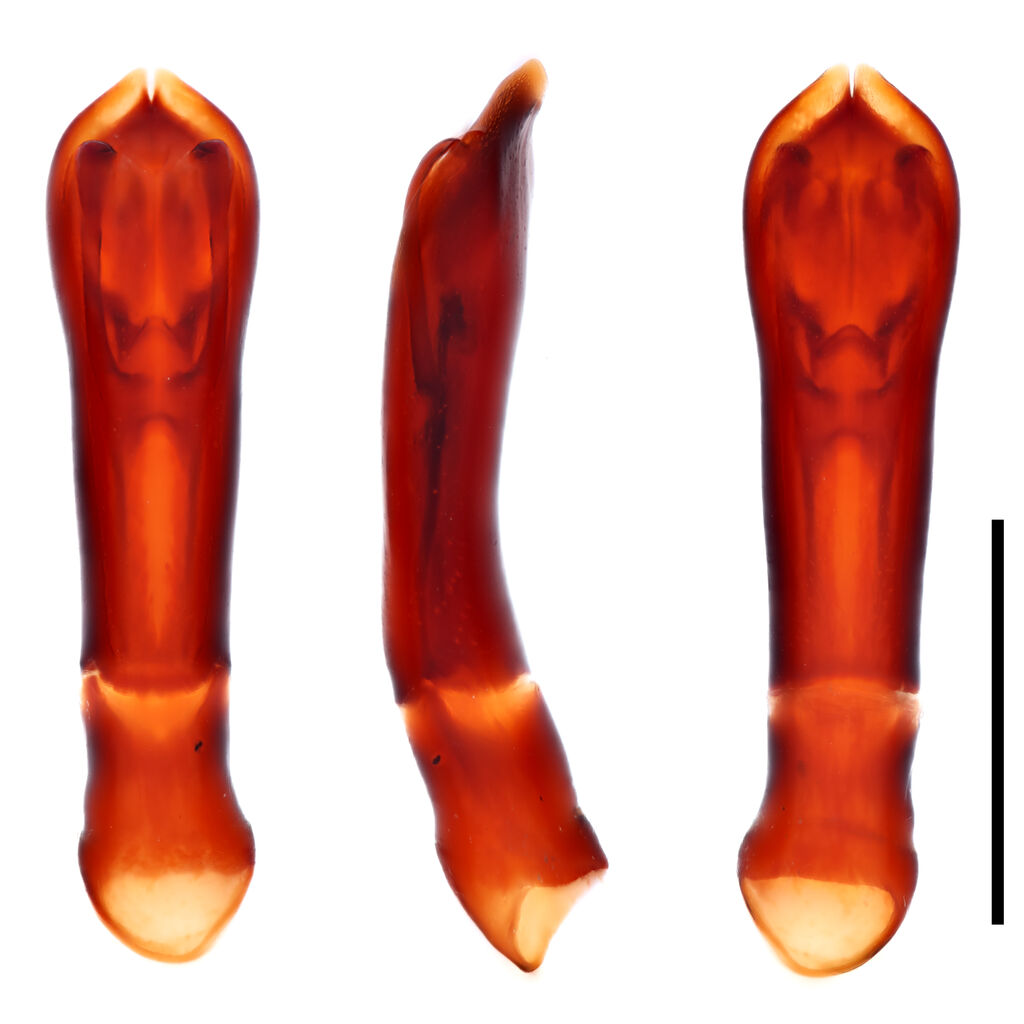
Aedeagus of *H.shanghaicus* Marseul, 1862 from dorsal, lateral and ventral view (left to right). Scale bar = 1 mm.

**Figure 3.**
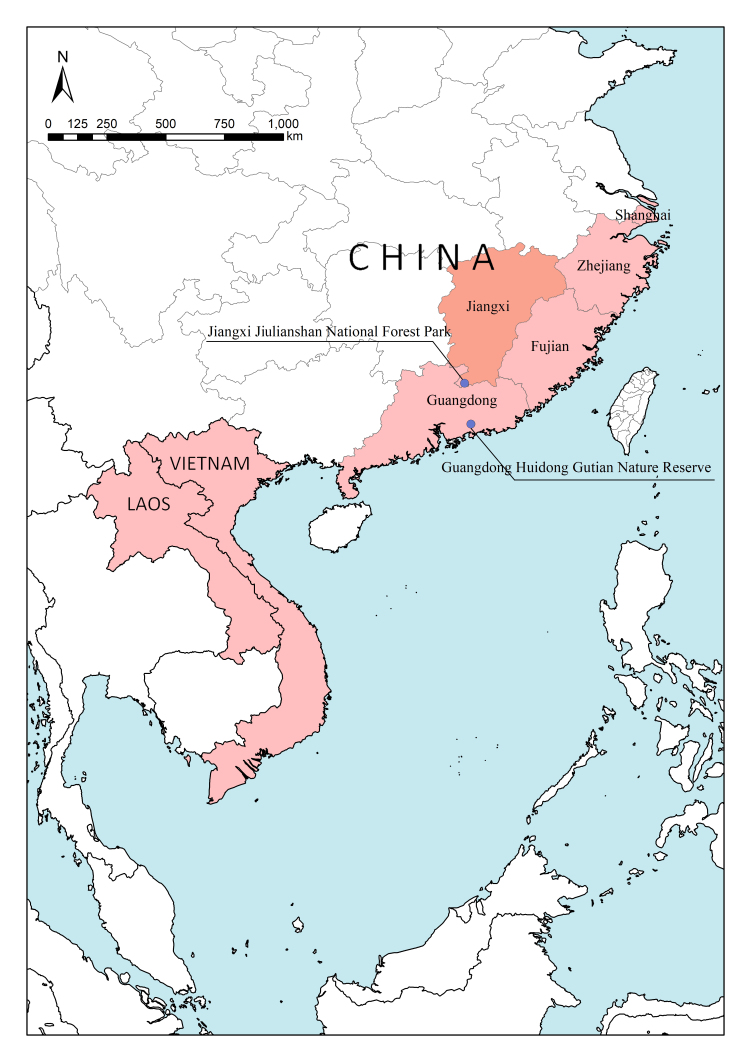
Distribution of *H.shanghaicus* Marseul, 1862: China (Shanghai, Zhejiang, Fujian, Guangdong, Jiangxi), Laos and Vietnam. (●) Collection locations: Gutian and Jiulianshan.

Specimens (Fig. 1) were identified using the key by Zhou et al. (2021). A total of 30 specimens, 15 from Gutian and 15 from Jiulianshan, were identified as *Histershanghaicus*. The identity of six male specimens, three from Gutian and three from Jiulianshan, has been confirmed by comparing their male genitalia (Fig. 2) with the drawings in Mazur (2011a). This is the first record of *H.shanghaicus* from Jiangxi Province, China (Fig. 3).

## Discussion

**Figure 4.**
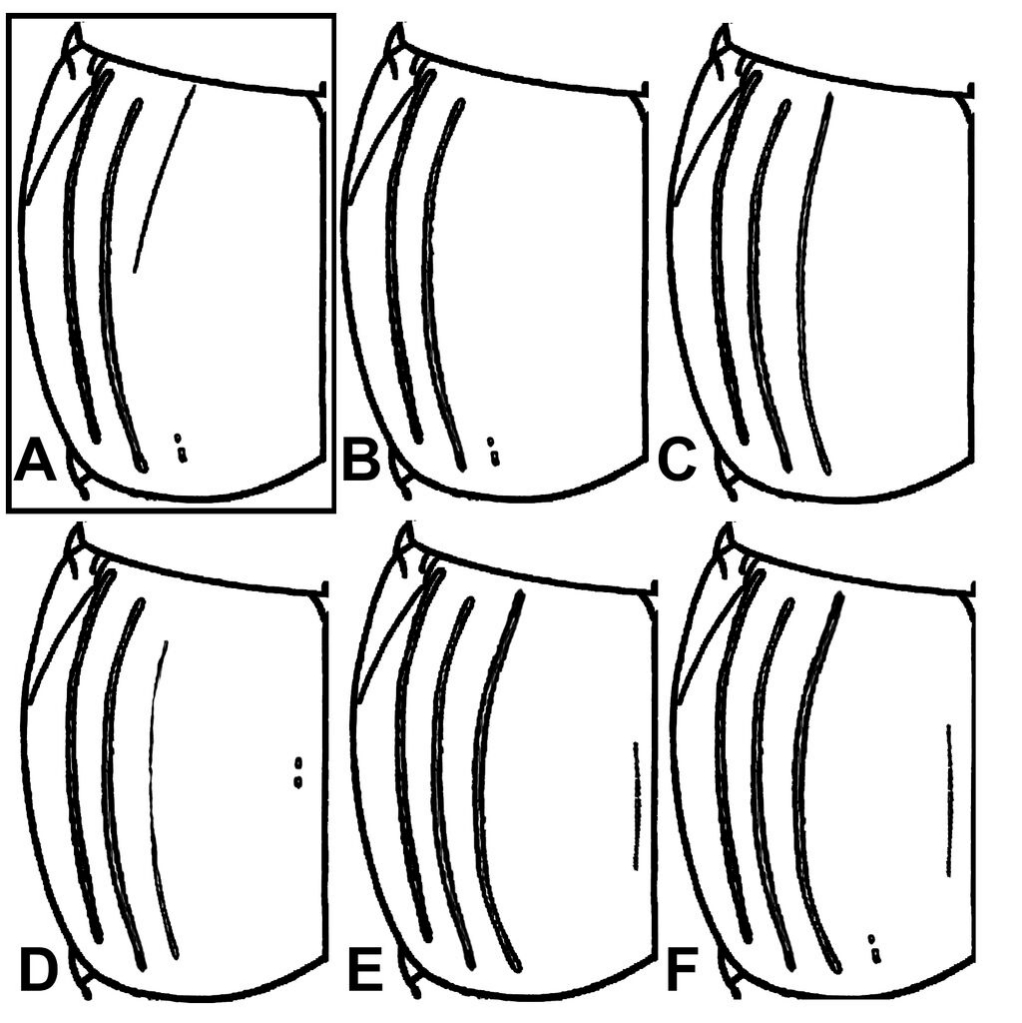
Dorsal elytral stria variation of *H.shanghaicus* Marseul 1862, with typical ‘*shanghaicus*’ framed.

**Figure 5.**
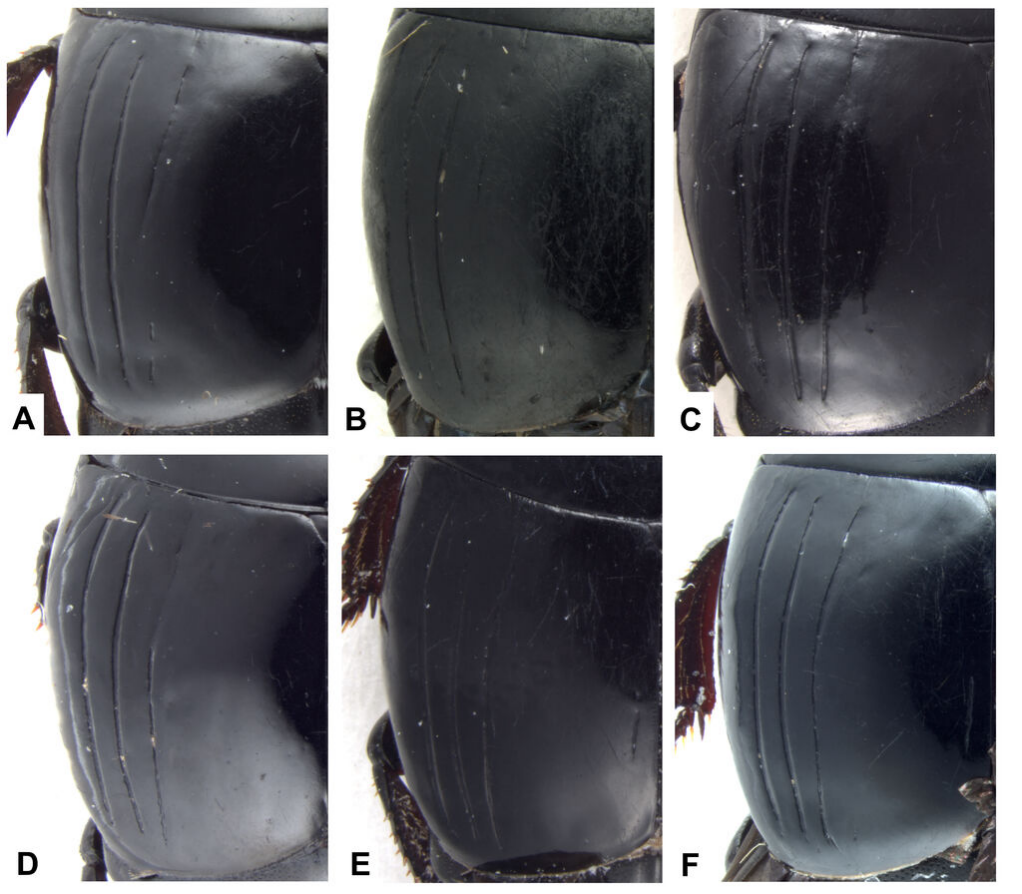
Dorsal elytral stria variations (photos, conjuction to Fig. 4) of *H.shanghaicus* Marseul, 1862.

A typical specimen of *H.shanghaicus* possesses dorsal elytral striae I–II complete and slightly basally abbreviated stria III. Normally, sutural elytral stria is absent. Our examination showed the variation in the configuration of the dorsal elytral striae in *H.shanghaicus* from both localities. The extremes range from fully developed dorsal elytral stria III (complete) with nearly complete sutural elytral stria, to complete dorsal elytral striae I–II (with stria III obsolete) with strongly reduced or absent sutural stria (Figs 4, 5).

Configuration of the dorsal elytral striae has historically played an active role in species descriptions; i.e. *Histerjavanicus* Paykull, 1811, possesses elytral striation that is highly variable and has resulted in seven synonymies of this species (Mazur 2011a, Mazur 2011b). *Histershanghaicus* and *H.borneensis* Desbordes, 1919 are the only two oriental species with the anterior mesoventral margin being almost straight and the apical protibial tooth bearing two spinules (Mazur 2011a). Although the elytral striation varies amongst *Histershanghaicus* specimens, the male genitalia remains a stable character for species identification. We were able to confirm the conspecificity of our specimens through comparative analysis between the images of the genitalia we took, with drawings of the same species by Mazur (2011a). Therefore, when in doubt in histerid taxonomy, we advocate for the examination of the male genitalia, which serves as a more reliable diagnostic character for the species in this genus.

## References

[B12793737] Bickhardt H (1910). Beiträge zur Kenntnis der Histeriden IV. Entomologischen Blättern.

[B12637097] Mazur S (1985). A new species of *Hister* and notices of others (Col. Histeridae). Revue Suisse de Zoologie.

[B12637106] Mazur Sławomir (2011). Review of the Oriental species of the genus *Hister* Linnaeus, 1758 (Coleoptera: Histeridae). Annales Zoologici.

[B12794721] Mazur S (2011). A concise catalogue of the Histeridae (Coleoptera).

[B12637133] Nie R, Yang M, Xue H (2017). The application and effectiveness of a flight interception trap for insect collecting. Journal of Applied Entomology.

[B12637124] Ôhara Masahiro (1989). Notes on six histerid beetles from Southern Asia (Coleoptera: Histeridae). Insecta Matsumurana.

[B12637115] Ôhara Masahiro (1994). Revision of the superfamily Histeroidea of Japan (Coleoptera).. Insecta Matsumurana.

[B12637142] Zhou H, Zhang Y, Luo T (2021). Fauna Sinica: Insecta. Volume 75. Coleoptera: Histeroidea, Sphaeritidae, Synteliidae and Histeridae.

